# Development of a colloidal gold immunochromatographic assay strip using monoclonal antibody for rapid detection of porcine deltacoronavirus

**DOI:** 10.3389/fmicb.2022.1074513

**Published:** 2023-01-05

**Authors:** Wei Wang, Baochao Fan, Xuehan Zhang, Rongli Guo, Yongxiang Zhao, Junming Zhou, Jinzhu Zhou, Qi Peng, Mingjun Zhu, Jizong Li, Bin Li

**Affiliations:** ^1^Institute of Veterinary Medicine, Jiangsu Academy of Agricultural Sciences, Key Laboratory of Veterinary Biological Engineering and Technology Ministry of Agriculture and Rural Affairs, Nanjing, China; ^2^Shaoxing Academy of Biomedicine of Zhejiang Sci-Tech University, Shaoxing, China; ^3^Jiangsu Key Laboratory for Food Quality and Safety, State Key Laboratory Cultivation Base of Ministry of Science and Technology, Nanjing, China; ^4^Jiangsu Co-Innovation Center for Prevention and Control of Important Animal Infectious Diseases and Zoonoses, Yangzhou University, Yangzhou, China; ^5^Key Laboratory for Prevention and Control of Avian Influenza and Other Major Poultry Diseases, Ministry of Agriculture and Rural Affairs, Guangzhou, China

**Keywords:** porcine deltacoronavirus, colloidal gold immunochromatographic assay (GICA) strip, monoclonal antibodies, real-time PCR, cross-species transmission

## Abstract

Porcine deltacoronavirus (PDCoV) cause diarrhea and dehydration in newborn piglets and has the potential for cross-species transmission. Rapid and early diagnosis is important for preventing and controlling infectious disease. In this study, two monoclonal antibodies (mAbs) were generated, which could specifically recognize recombinant PDCoV nucleocapsid (rPDCoV-N) protein. A colloidal gold immunochromatographic assay (GICA) strip using these mAbs was developed to detect PDCoV antigens within 15 min. Results showed that the detection limit of the GICA strip developed in this study was 10^3^ TCID_50_/ml for the suspension of virus-infected cell culture and 0.125 μg/ml for rPDCoV-N protein, respectively. Besides, the GICA strip showed high specificity with no cross-reactivity with other porcine pathogenic viruses. Three hundred and twenty-five fecal samples were detected for PDCoV using the GICA strip and reverse transcription-quantitative real-time PCR (RT-qPCR). The coincidence rate of the GICA strip and RT-qPCR was 96.9%. The GICA strip had a diagnostic sensitivity of 88.9% and diagnostic specificity of 98.5%. The specific and efficient detection by the strip provides a convenient, rapid, easy to use and valuable diagnostic tool for PDCoV under laboratory and field conditions.

## 1. Introduction

Porcine deltacoronavirus (PDCoV), which belongs to the genus *Deltacoronavirus* in the family *Coronaviridae* of the order *Nidovirales* (Walker et al., 2019), is a emerging swine enteropathogenic coronavirus that causes acute diarrhea, vomiting, and dehydration in newborn piglets (Chen et al., 2015; Vitosh-Sillman et al., 2016). PDCoV was initially reported in Hong Kong during a territory-wide molecular epidemiology study in mammals and birds in 2012 (Woo et al., 2012). Subsequently, in early 2014, the first outbreak of PDCoV-associated diarrhea was emerged in swine in Ohio (United States; Wang et al., 2014a) and then spread to other US states (Wang et al., 2014b). Subsequently, the virus has been detected in fecal samples from piglets in Canada (Marthaler et al., 2014), South Korea (Lee et al., 2016), Japan (Suzuki et al., 2018), Thailand (Lorsirigool et al., 2017), Vietnam (Saeng-Chuto et al., 2017), and Lao PDR (Lorsirigool et al., 2016). In 2014, PDCoV was first detected in domestic pigs in mainland China (Zhao et al., 2017). Even independent infections of PDCoV among Haitian children have been reported (Lednicky et al., 2021). Experimental infection studies showed that calves, chickens, turkey poults, mice are susceptible to infection with PDCoV, standing for its potential for cross-species transmission (Woo et al., 2012; Duan, 2021). The PDCoV outbreak has exhibited a global spread and caused significant economic losses in pig industry worldwide.

The complete genome of PDCoV is approximately 25.4 kb in length (Zhang et al., 2019; Tang et al., 2021), making it the smallest genome known among Coronaviruses (CoVs). The genome arrangements of PDCoV are as follows: 5’UTR-ORF1a-ORF1b-S-E-M-NS6-N-NS7-3’UTR (Duan, 2021; Jin et al., 2021; Tang et al., 2021). ORF1a and ORF1b occupy the 5′-proximal two-thirds of the complete genome and code for two overlapping replicase precursor polyproteins, pp1a and pp1ab, which are cleaved into non-structural proteins which involved in viral replication and transcription. The 3′-proximal last third of the genome encodes four structural proteins (S, E, M and N), and at least three nonstructural proteins (NS6, NS7 and NS7a; Zhang et al., 2019; Duan, 2021; Jin et al., 2021). The N protein is a highly immunogenic protein and the most abundant viral protein expressed in virus-infected cells, which makes it a suitable candidate for the detection of virus-specific antibodies and disease diagnosis (Wang et al., 2019; Tang et al., 2021).

The epidemiological, clinical, and pathological features are similar among PDCoV, porcine epidemic diarrhea virus (PEDV) and transmissible gastroenteritis virus (TGEV; Ding et al., 2020; Tang et al., 2021), leading to difficulties in the clinical differential diagnosis. Although several detection methods, including virus neutralization tests, virus isolation, and indirect immunofluorescence assay (IFA), are available for the detection of viruses, these methods are not applicable for detection in large-scale samples and point-of-care testing (POCT; Zhang, 2016; Ding et al., 2020). Currently, reverse transcriptase real-time PCR (RT-qPCR; Pan et al., 2020) or RT-PCR (Wang et al., 2014a; Ding et al., 2020) assays and sandwich enzyme-linked immunosorbent assay (ELISA; Wang et al., 2021) for PDCoV detection have been reported. However, these methods are labor-intensive and time-consuming, also requiring qualified personnel and appropriate biosafety facilities.

Colloidal gold immunochromatographic assay (GICA) is a highly useful tool in diagnostics based on the specific antigen–antibody immunoreactions, and has been successfully used for rapidly detection in kinds of samples especially specific antigens or antibodies of multiple diseases (Sheng et al., 2012; Liu et al., 2021). Compared with other laboratory-based diagnostic platform analyses, the assay results are directly visible to the naked eye, and without requiring specialized equipment, untrained personnel, and complicated handling procedures, which provide convenience for rapid testing. However, the GICA strip for detection of PDCoV has not been described. So, this study aimed to establish a GICA-based test strip as a supplementary technique for rapidly detecting PDCoV in fecal samples from pigs. This method was simple, rapid, and specific for detecting PDCoV, which is suitable for pathogen detection in laboratory and clinical samples.

## 2. Materials and methods

### 2.1. Viruses and cell culture

PDCoV CZ2020 strain (GenBank accession number: OK546242) was isolated and maintained in our laboratory. The LLC-PK1 cell line was cultured in Dulbecco’s Modified Eagle’s Medium (DMEM; Thermo Fisher Scientific, MA, United States) supplemented with 10% heat-inactivated fetal bovine serum (FBS; Tianhang, China) and antibiotics (0.25 μg/ml of amphotericin B, 100 μg/ml of streptomycin, and 100 U/ml of penicillin; Thermo Fisher Scientific). LLC-PK1 cells were purchased from the China Institute of Veterinary Drug Control, which maintained in DMEM (containing 7.5 μg/ml trypsin) and used to propagate PDCoV. When cytopathic effects (CPE) were observed (over 85% cells were split), the infected cell cultures were collected and freeze-thawed, and cell debris was removed by centrifugating at 4,000 ×*g* at 4°C for 10 min. The supernatant were collected and stored at −80°C until used.

PEDV/AH2010 (The virus was cultured in Vero cells and titer was 10^6.5^ TCID_50_/ml), TGEV/JS2012 (The virus was cultured in ST cells and titer was 10^8.0^ TCID_50_/ml), porcine rotavirus (PoRV/NING86 was cultured in Marc145 cells and titer was 10^7.5^ TCID_50_/ml), porcine reproductive and respiratory syndrome virus (PRRSV/NF was cultured in Marc145 cells and titer was 10^6.0^ TCID_50_/ml), classical swine fever virus (CSFV/C was cultured in ST cells and titer was 10^6.0^ TCID_50_/ml), porcine circovirus type 2 (PCV2/2010AHCY was cultured in PK15 cells and titer was 10^7.0^ TCID_50_/ml), and pseudorabies virus (PRV/AH02LA was cultured in ST cells and titer was 10^8.0^ TCID_50_/ml) were conserved in the laboratory. PEDV/AH2010, TGEV/JS2012, PoRV/NING86, PRRSV/NF and PCV2/2010AHCY were isolated in our lab. CSFV/C was obtained from commercial vaccine. PRV/AH02LA was obtained from Jichun Wang’s lab of Institute of Veterinary Immunology and Engineering, JAAS. Besides, the titer of these viruses had been detected to make sure these viruses were present and enough viral load for using to analyse the specificity of the GICA strip.

### 2.2. Preparation of monoclonal antibody and rPDCoV-N protein

rPDCoV-N protein and two monoclonal antibodies (mAb-32^#^ and mAb-33^#^) against the protein were prepared according to our previous study (Wang et al., 2021), and the two mAbs were identified by western blot and IFA in our laboratory.

Following the procedures described previously with slight modifications (Wang et al., 2021), the purified rPDCoV-N protein were separated by SDS-PAGE and transferred to PVDF membranes using a Bio-Rad Mini Trans-Blot Cell (Bio-Rad). The membranes were, respectively, incubated with mAb-32^#^ (5.1 μg/ml for final concentration) or mAb-33^#^ (3.9 μg/ml for final concentration) against PDCoV, followed by goat anti-mouse serum conjugated to horseradish peroxidase (HRP, 1:5000), and the target protein was visualized by enhanced chemiluminescence (ECL).

Indirect IFA was performed as described previously with slight modifications (Yu et al., 2019). Briefly, 10^7.0^ TCID_50_ /ml of PDCoV CZ2020 strain was diluted into 10^4.0^ TCID_50_ /ml with DMEM (7.5 μg/ml trypsin). Then, 500 μl of 10^4.0^ TCID_50_/ml PDCoV was inoculated into LLC-PK1 cells (approximately 90, % confluent) cultured in 24-well plates, and the virus was adsorbed for 2 h. Subsequently, the liquid of the plates was discarded, and the plates were washed twice with DMEM (7.5 μg/ml trypsin). Finally, 1 ml DMEM (7.5 μg/ml trypsin) was added to each plate. Twelve hours post-inoculation, the cells were washed twice with PBS, fixed with methyl alcohol for 1 h at 4°C, then blocked with 5% skim milk (in PBS) for 2 h at 4°C, and subsequently incubated with mAb-32^#^ (10.2 μg/ml for final concentration) or mAb-33^#^ (7.8 μg/ml for final concentration) for 1 h at 37°C. Cells were washed thrice with PBST and incubated with goat anti-mouse IgG conjugated with FITC (Boster, China; 1:500) for additional 1 h at 37°C. Finally, the cells were washed thrice with PBST and observed under a fluorescence microscope (Olympus IX-51). Uninfected cells served as negative control.

### 2.3. Synthesis of colloidal gold

To prepare colloidal gold, 1 ml of 1% chloroauric acid (HAuCl_4_) was added to the Erlenmeyer flask with 99 ml ddH_2_O which was stirred and heating to boiling for 2 min. Then 2 ml of 1% sodium citrate aqueous solution was added accurately under constant agitation, followed to boiling for another 10 min. The colloidal gold suspension was cooled down to room temperature, and volume was fixed to 100 ml by adding ddH_2_O.

### 2.4. Preparation of the GICA strip

As previously described (Zhang et al., 2008; Lin et al., 2011), a colloidal gold solution was prepared. The colloidal gold solution was adjusted to pH 7.0 with potassium carbonate (K_2_CO_3_, 0.2 M) to prepare the detector reagent. The mAb-32^#^ was coupled to colloidal gold particles as previously described (Zeng et al., 2019; Liu et al., 2021). Briefly, purified mAb-32^#^ (45 μg/ml) was added to 1 ml of a 40 nm colloidal gold solution with gentle stirring. After 40 min, 10% bovine serum albumin (BSA) in PBS (w/v) was added to a final concentration of 0.2% and the solution was stabilized for 30 min. The solution was then centrifuged at 8,500 ×*g* at 4°C for 10 min and the soft pellet was resuspended with PBS (0.02 M, pH 7.4) containing 1.0% BSA. The resuspended solution was stored at 4°C.

**Figure 1:**
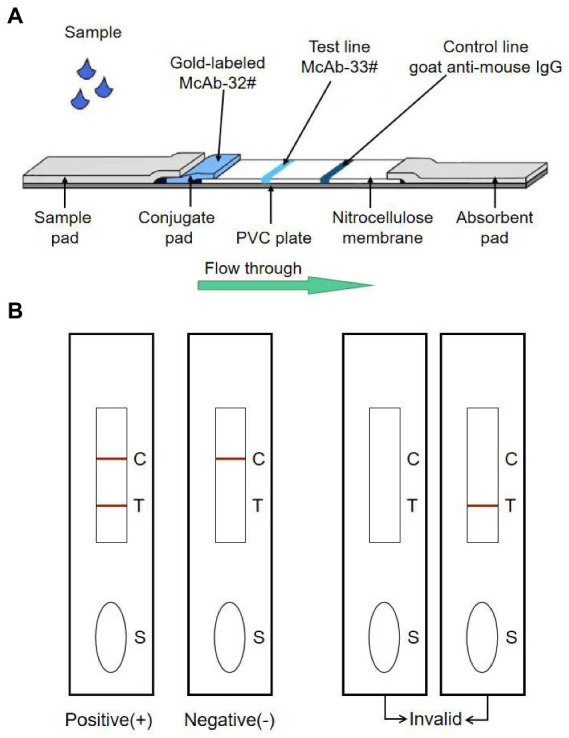
The schematic representation of the GICA strip. **(A)** The strip included three pads (sample, conjugate and absorbent), an NC membrane, and a PVC plate. The conjugate pad contained the dried gold-labeled mAb-32^#^, which provided an easily visible red color. There were two lines on the NC membrane: the control line and the test line. The test line contained mAb-33^#^. The control line contained the goat anti-mouse IgG antibody. **(B)** The detecting principle of the GICA strip.

The immunochromatography strip was constructed as in previously studies (Xu et al., 2010; Lin et al., 2011; Yu et al., 2019). Colloidal gold-labeled antibody conjugate was jetted onto glass fiber and dried at 37°C. Goat anti-mouse IgG antibody (1.0 mg/ml) was dispensed onto a nitrocellulose (NC) membrane on the upper line (C line) for control with a volume of 1 μl per 1 cm line, and for another epi-position strain mAb-33^#^ (1.0 mg/ml) in PBS was jetted into the lower part for test line (T line); the dispensed volume was also of 1 μl per 1 cm line. The remaining active sites on the membrane were blocked by incubation with 2% BSA in PBS (1 ml/cm membrane) for 30 min at room temperature. The membrane was washed once with PBS and again with ddH_2_O and then, dried at 37°C. Finally, the sample pad, pre-treated conjugate pad, NC membrane, and absorbent pad adhered to a plate in the proper order, which was subsequently cut into 0.3 cm × 6 cm strips (Figure 1A).

### 2.5. Sensitivity of the GICA strip

To evaluate the sensitivity of the GICA strip, the PDCoV CZ2020 strain cell culture virus (10^7^ TCID_50_/ml) was serially diluted to 10^6^, 10^5^, 10^4^, 10^3^, 10^2^, 10 and 1 TCID_50_/ml by using PBS. Then these samples were detected using the strips and RT-qPCR. Otherwise, different concentrations of purified rPDCoV-N protein (diluted to 5.0, 1.0, 0.5, 0.25, 0.125, 0.0625 and 0.0313 μg/ml by using PBS) were tested using the strips. PBS and DMEM (containing 7.5 μg/ml trypsin and 10% FBS) were used as blank controls. Approximately 100 μl of sample was added to the sample pad and waiting for 15 min. When red-purple bands appeared at both the test and control lines, the result was considered positive. When a red-purple band only appeared at the control line, the result was considered negative (Figure 1B). The same procedure was repeated 3 times with different operators.

The RNA of PDCoV serially diluted samples and blank controls were extracted, and cDNA was synthesized by commercial kits (HiScript II Q RT SuperMix for qPCR, Vazyme, China). Then the cDNA of these samples was detected by qPCR. The qPCR primers of PDCoV M gene (forward, ATCGACCACATGGCTCCAA; reverse primer, CAGCTCTTGCCCATGTAGCTT) and a probe (FAM-CACACCAGTCGTTAAGCATGGCAAGCT-BHQ1) was run on QuantStudio 6 Real-Time PCR Systerm (ThermoFisher, Carlsbad, CA, United States) with the following conditions: 5 min at 95°C, followed by 40 cycles of 10 s at 95°C and 30 s at 60°C. Assign a cycle threshold (C_t_) value to each PCR reaction from a scan of all amplification plots (a plot of the fluorescence signal versus cycle number). If test samples have a C_t_ value ≥35.0, it is considered the samples are negative; and if test samples have a C_t_ value <35.0, it means the samples are positive (strongly positive samples have a C_t_ value <25.0).

### 2.6. Specificity of the GICA strip

PEDV, TGEV, PoRV, PRRSV, CSFV, PCV2 and PRV were tested with the strip to evaluate the specificity of the GICA strip. PDCoV CZ2020 strain cell culture supernatant and LLC-PK1 cells were detected as positive and negative control, respectively.

### 2.7. Comparison of the GICA strip and RT-qPCR in clinical field samples detection

A total of 325 fecal samples obtained from different swine farms (Table 1) were examined by using the GICA strip and RT-qPCR. The fecal swabs were stirred into PBS solution, and then stood for 1–2 min. The diagnostic sensitivity, specificity and accuracy were calculated using the following formulas: diagnostic sensitivity = true positive/(true positive + false negative) × 100%; diagnostic specificity = true negative/(true negative + false positive) × 100%; consistency = (true positive + true negative)/(true positive + false positive + true negative + false negative) × 100%. The agreement between the GICA strip and RT-qPCR was measured with the kappa statistic value (Tang et al., 2015).

**Table 1:** The information on clinical field samples from swine farms.

The position of swine farms	Amount of fecal samples	Symptoms of neonatal piglets
Taian, Shangdong	50	Diarrhea
Yancheng, Jiangsu	82	Diarrhea
Huaian, Jiangsu	86	Diarrhea and vomiting
Taizhou, Jiangsu	50	Diarrhea
Yixing, Jiangsu	57	Diarrhea and vomiting

### 2.8. Ethics statement

All applicable international, national, and/or institutional guidelines for the care and use of animals were followed by the Jiangsu Academy of Agricultural Sciences Experimental Animal Ethics Committee (NKYVET 2015-0127).

## 3. Results

### 3.1. Identification of mAbs

**Figure 2:**
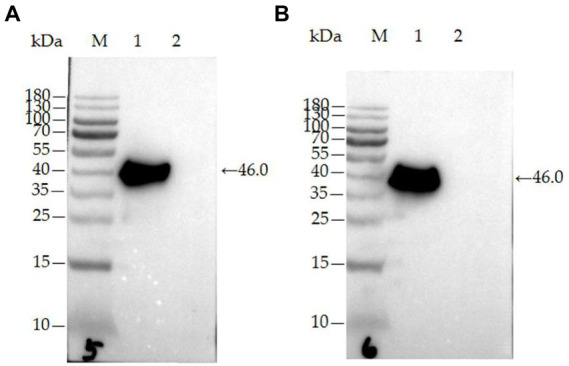
Characterization of mAb 32^#^ and 33^#^ by western blot. **(A)** mAb-32^#^; **(B)** mAb-33^#^. M-MW markers, 1-the purified rPDCoV-N protein, 2-the whole cell lysate without induction.

**Figure 3:**
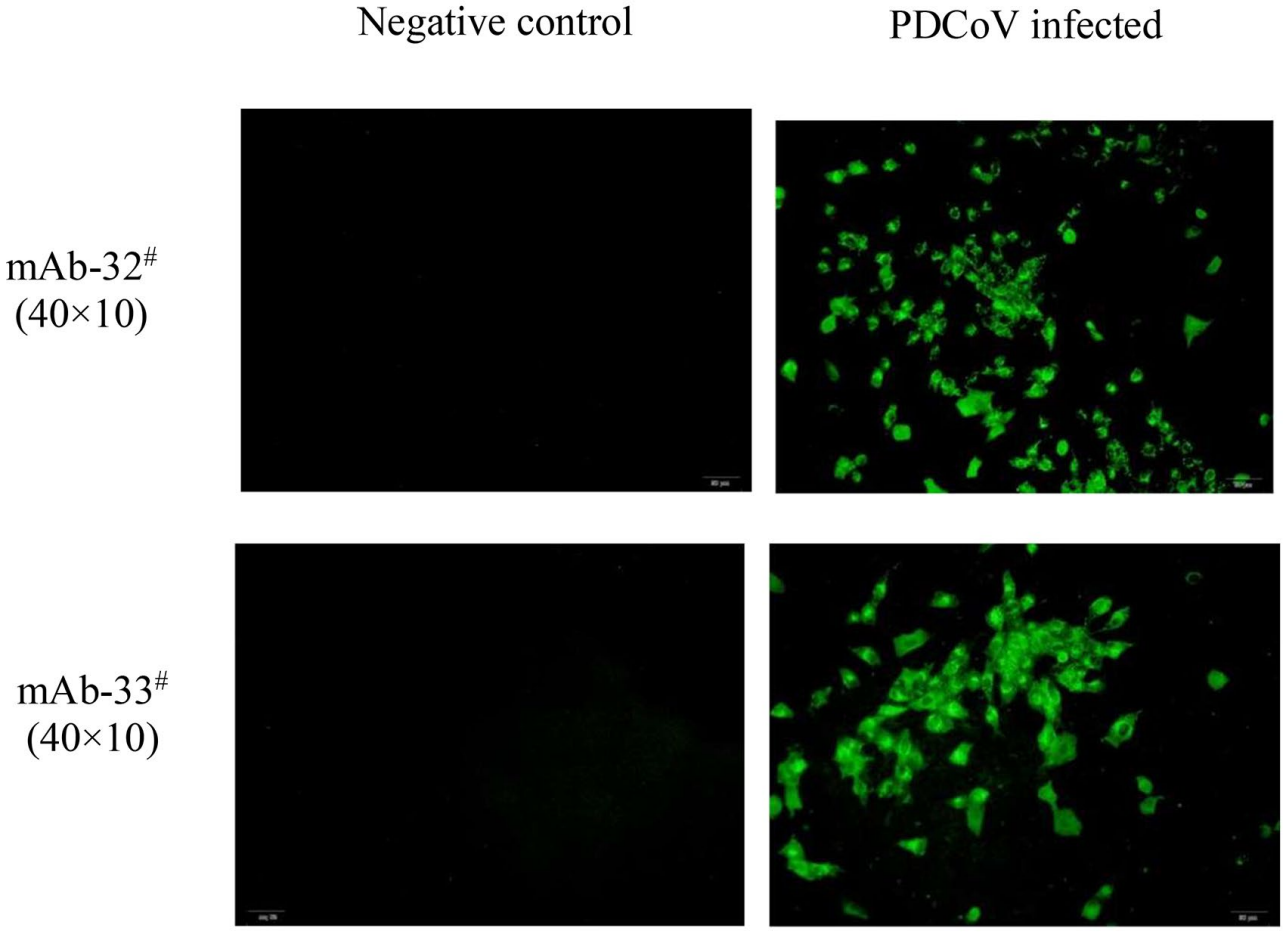
IFA analysis of mAb 32^#^ and 33^#^. Both antibodies recognized the nucleocapsid protein in PDCoV-infected LLC-PK1 cells. The uninfected LLC-PK1 cells were used as a negative control.

The two mAbs were identified by western blot and IFA. Purified rPDCoV-N proteins were subjected to western blot analysis, and the results demonstrated that the two mAbs could recognize the nucleocapsid protein (approximately 46.0 kDa) of PDCoV (Figures 2A,B). IFA showed that the mAbs could specifically react with PDCoV (Figure 3), thus indicating that the two mAbs are applicable for developing diagnostic methods to detect PDCoV antigens.

### 3.2. Sensitivity of the GICA strip

**Figure 4:**
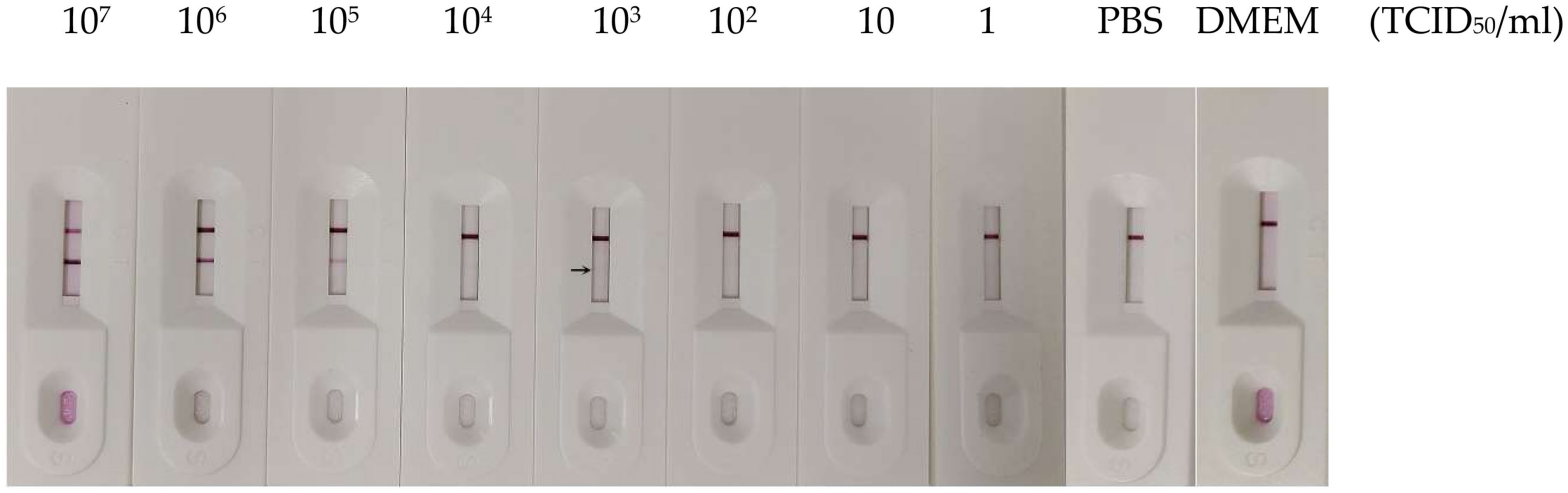
Sensitivity of the GICA strip for detecting PDCoV. Different virus titers of PDCoV CZ2020 strain were detected by the strip. PBS and DMEM were used as the negative control.

**Figure 5:**
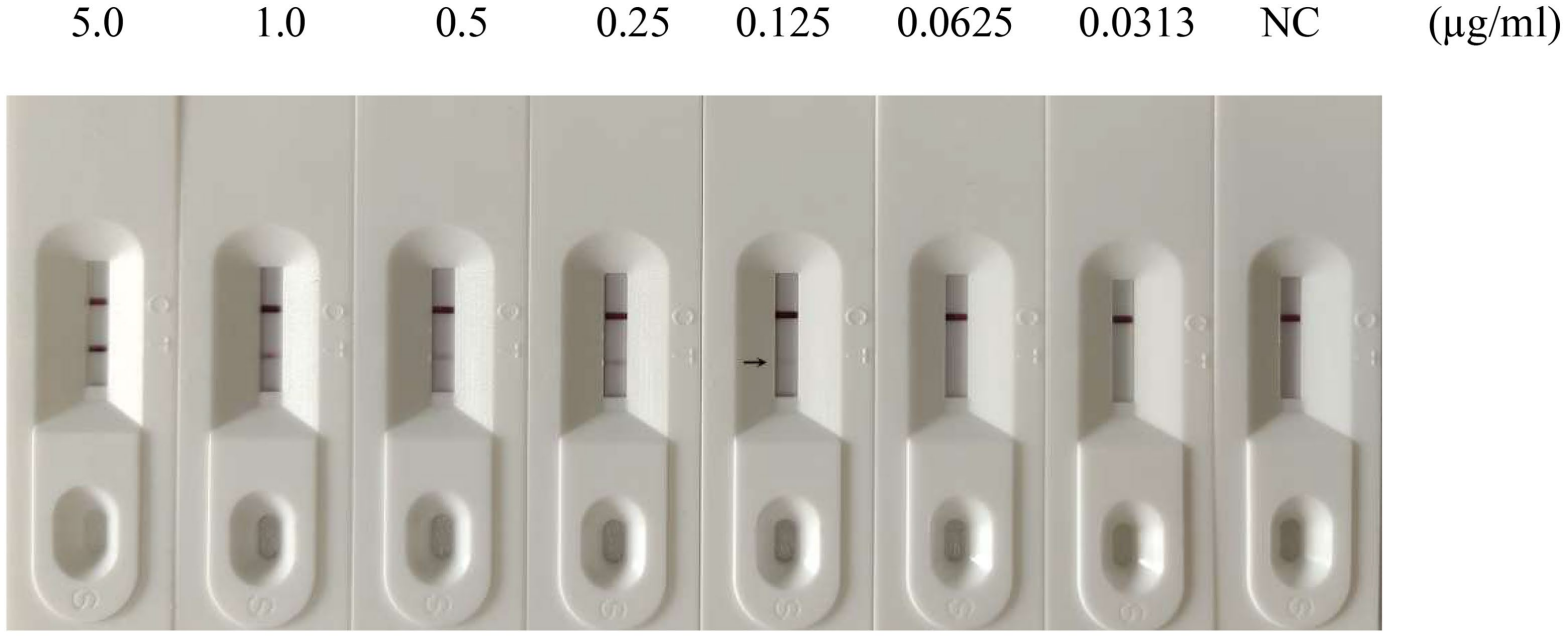
Sensitivity of the GICA strip for detecting rPDCoV-N protein. Different concentrations of the protein (5.0, 1.0, 0.5, 0.25, 0.125, 0.0625 and 0.0313 μg/ml) were detected by the strip. PBS was used as the negative control (NC).

To evaluate the sensitivity of the GICA strip, the assay’s detection limit was determined by testing against dilutions of PDCoV CZ2020 strain and rPDCoV-N protein. Results of chromogenic reaction revealed that the strip was able to detect PDCoV CZ2020 strain at a level of 10^3^ TCID_50_/ml (Figure 4) and rPDCoV-N protein at a level of 0.125 μg/ml (Figure 5). In parallel, the RT-qPCR assay detected the viral genome at a limit of 10^2^ TCID_50_/ml (Table 2), which was 10-fold more sensitive than the GICA strip.

**Table 2:** Sensitivity of the qPCR for detecting PDCoV.

Samples	PDCoV CZ2020 strain (TCID_50_/ml)								Negative control	
	10^7^	10^6^	10^5^	10^4^	10^3^	10^2^	10	1	PBS	DMEM
C_t_ value	15.95	19.66	23.05	26.5	30.81	33.3	36.91	Undetermined	Undetermined	Undetermined
Determination of results	++	++	++	+	+	+	−	−	−	−

“++” indicates strongly positive results, and “+” indicates the positive results by RT-qPCR, while “−” indicates the negative results.

### 3.3. Specificity of the GICA strip

**Figure 6:**
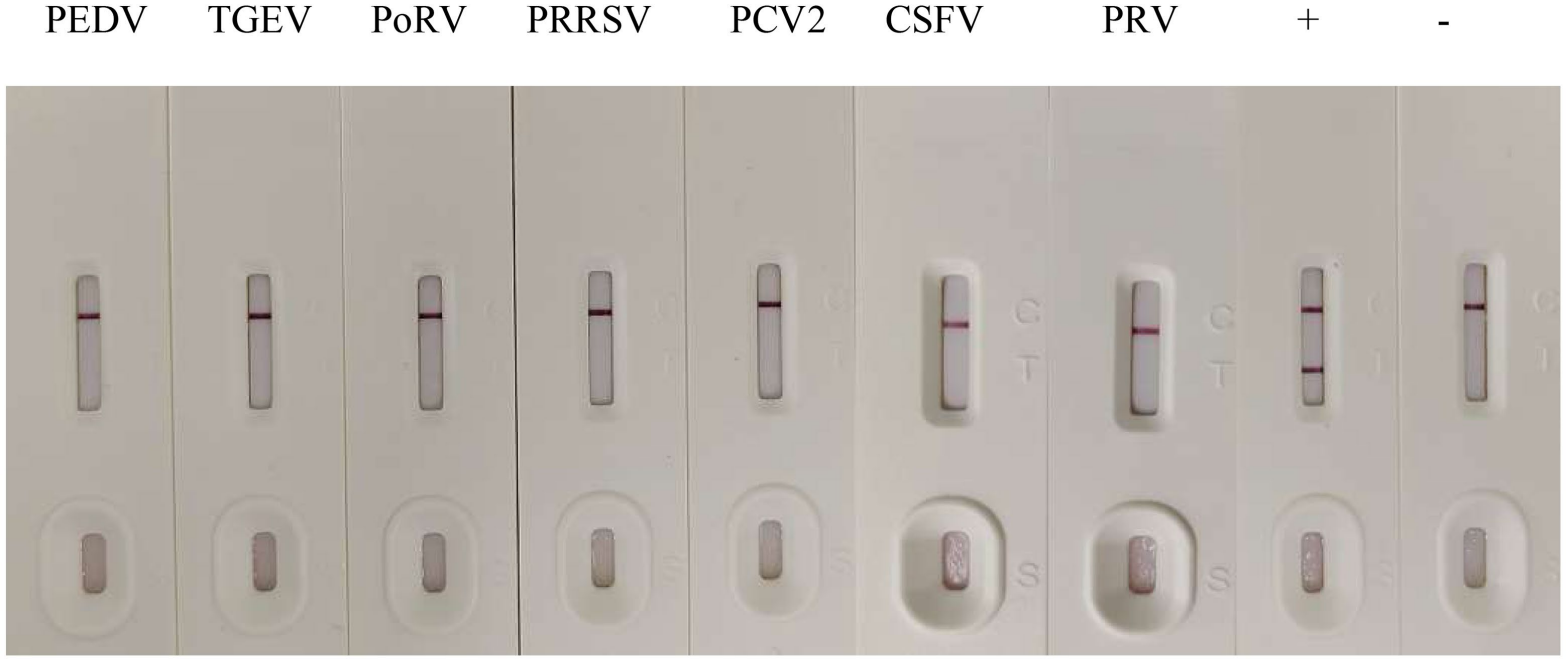
Specificity of the GICA strip. PDCoV cell culture supernatant as a positive control (+), LLC-PK1 cells as negative control (−), PEDV, TGEV, PoRV, PRRSV, PCV2, CSFV and PRV were tested with the strip.

The specificity of the GICA strip was evaluated using common swine pathogens, such as PEDV, TGEV, PoRV, PRRSV, PCV2, CSFV and PRV. While PDCoV cell culture supernatant yielded positive result, all other samples showed negative results (Figure 6). These data convincingly demonstrated that the strip could be used to detect PDCoV specifically.

### 3.4. Clinical field samples detection

A total of 325 fecal samples were examined by using the GICA strip and RT-qPCR (Table 3). The GICA strip was found to have 88.9% diagnostic sensitivity [48/(48 + 6)] and 98.5% diagnostic specificity [267/(4 + 267)] relative to RT-qPCR. The consistency of these two detection methods was [(48 + 267)/(52 + 273)] = 96.9%. An example of detection of a fecal sample using GIGA strip is shown in Supplementary Figure S1. No bands were identified at low virus titers, but bands were detected at high virus titers. In addition, the kappa value was 0.887, which is considered ‘almost perfect’ agreement between the two detection methods. The positive rate of PDCoV using the GICA strip was (48 + 4)/325 = 16.0% versus (48 + 6)/325 = 16.6% detected by RT-qPCR.

**Table 3:** Comparison of RT-qPCR and the GICA strip for detecting PDCoV in fecal samples.

Fecal samples		GICA strip			Kappa value
		Positive	Negative	Total	
RT-qPCR	Positive	48	6	54	0.887
	Negative	4	267	271	
	Total	52	273	325	

This result showed that PDCoV infection had been become one of swine farm’s most important enteropathogenic pathogens. Also, these results show it is a good agreement for PDCoV detection between the GICA strip and RT-qPCR, and the developed strip would be effective in rapidly identifying of PDCoV antigens in fecal samples from swine farms.

## 4. Discussion

Coronaviruses (CoVs) are existed widely among mammals and birds (Tang et al., 2021). As globally important pathogens, zoonotic CoVs have a higher risk for cross-species transmission to humans and animals (Thakor et al., 2022). We found that PDCoV can infect swines of different ages, while piglets are more susceptible. In experimental infection researches, we also confirmed that calves, chickens, mice, turkey poults are susceptible to infecting PDCoV (Duan, 2021). Even in November 2021, Lednicky et al. (2021) first reported that cross-species transmission of PDCoV may have occurred from swines to children in Haiti. It has been posing a threat to the swine population and persons with direct exposure to pigs (e.g., pig farm workers and slaughterhouse workers). Besides, PDCoV infections have resulted in economic losses for the global swine industry (Ma et al., 2015; Zhang et al., 2020). So, rapid and early diagnosis is crucial to prevent and control PDCoV for swine health.

Currently, many methods for PDCoV detection have been developed, which were divided into serological and virological methods. Common virological methods include the detection of viral nucleic acid (various RT-PCRs (Marthaler et al., 2014; Wang et al., 2014b) and *in situ* hybridization (Jung et al., 2015)), viral antigen (immunofluorescence staining (Chen et al., 2015; Zhang et al., 2020), immunohistochemistry (Ma et al., 2015; Zhang et al., 2020) and sandwich ELISA (Wang et al., 2021)), virus particles (electron microscopy (Ma et al., 2015)) and virus isolation (Ma et al., 2015). The most commonly used serological assays include virus neutralization test (VNT; Zhang et al., 2020) and ELISA (Su et al., 2016; Zhang et al., 2020). However, these assays require to spend several hours even several days and need qualified personnel or expensive specialized equipment, which is often unaffordable for the mass detection in swine farms, specially POCT. To detect PDCoV from fecal samples in lesser time and achieve the control of this disease in swine farms, we have developed an antigen-capture colloidal GICA strip method, based on the use of a mAb conjugated with colloidal gold particles, which do not require special training or tools and yields rapid results within 15 min. The virus detection capacity of the GICA strip was systematically evaluated in this study, and all the obtained results suggested that the strip was a convenient method to detect and control the PDCoV infection.

To get a more specific and sensitive GICA strip, we first systematically studied the characterization of two mAbs (32^#^, 33^#^) by western blot and IFA. Then, the reaction conditions of the GICA strip were optimized, including the pH of the colloidal gold fluid, the amount of labeled mAb-33^#^ used, and the concentrations of colloidal gold-mAb-32^#^ conjugate and goat anti-mouse IgG (Data not shown). After optimization, the GICA strip gave an accurate and clear result, visualized within 15 min by the naked eye. We further examined the accuracy of the result, including specificity, sensitivity, and coincidence rate with RT-qPCR.

During the sensitivity evaluation, the GICA strip detected PDCoV at 10^3^ TCID_50_/ml (C_t_ value is 30.81 by RT-qPCR), whereas RT-qPCR could detect 100 TCID_50_/ml (C_t_ is 33.30 by RT-qPCR). Although the sensitivity of the GICA strip was lower than that of RT-qPCR for the detection of clinical samples, the coincidence rates with RT-qPCR were confirmed to be over 96%. The data suggested that the GICA strip could detect PDCoV in fecal samples effectively.

The GICA strip was used to detect PDCoV in 325 clinical fecal samples to examine its practicability. Among them, the results obtained from the strip agreed with RT-qPCR up to 96.9%. Eight samples which were identified as positive by RT-qPCR but missed by the GICA strip. These results are attributed to the excessively low virus content in the samples. Five other samples were PDCoV-negative by RT-qPCR but PDCoV-positive by the GICA strip. The reason of this disagreement might be PCR-suppression effect and degradation of nucleic acids in assays, which affected the accuracy of qPCR. This finding suggests that the developed strips effectively identify PDCoV in swine farms.

PDCoV was often involved in co-infection with other porcine viruses in previous studies (Zhang, 2016), such as PEDV (Song et al., 2015) and TGEV (Fang et al., 2021). Seven different DNA or RNA porcine viruses were used in this study to evaluate the specificity of the GICA strip. It showed that the strips were positive only for PDCoV cell culture supernatant, which indicated that the strips could be used to differentiate PDCoV from other porcine viruses, including PEDV, TGEV, PoRV, PRRSV, PCV2, CSFV and PRV.

In summary, the GICA strip developed in this study represents a means for the rapid and inexpensive detection of viral antigens to confirm PDCoV infection. The GICA strip exhibited high coincidence rates compared to RT-qPCR while taking only 15 min to yield results, which would allow a rapid diagnosis and early control of the disease.

## Data availability statement

The datasets presented in this study can be found in online repositories. The names of the repository/repositories and accession number(s) can be found at: https://www.ncbi.nlm.nih.gov/genbank/, OK546242.

## Ethics statement

The animal study was reviewed and approved by the Jiangsu Academy of Agricultural Sciences Experimental Animal Ethics Committee (NKYVET 2015-0127).

## Author contributions

WW took part in all the experiments and wrote the manuscript. BF helped to design the whole project and draft the manuscript. RG, YZ, and JiZ conducted cell culture, virus proliferation, RNA isolation and RT-qPCR detection. JuZ and QP conducted data analysis. XZ and MZ revised the English language of this article. BL and JL contributed essential ideas and discussion. All authors contributed to the article and approved the submitted version.

## Funding

This study was funded by National Key Research and Development Program (2022YFD1800601), National Natural Science Foundation of China (31802167, 31872481, 31941013, and 32002283), Jiangsu province Natural Sciences Foundation (BK20190003, BK20221432, and BK20210158), Jiangsu Agricultural Science and Technology Innovation Fund (CX(22)3028), the Special Project of Northern Jiangsu (SZ-LYG202109), China Postdoctoral Science Foundation (grant nos. 2022M711398 and 2022M711399), Open Fund of Shaoxing Academy of Biomedicine of Zhejiang Sci-Tech University (SXAB202215), Open Fund of Key Laboratory for prevention and control of Avian Influenza and Other Major Poultry Diseases, Ministry of Agriculture and Rural Affairs (YDWS202213).

## Conflict of interest

The authors declare that the research was conducted in the absence of any commercial or financial relationships that could be construed as a potential conflict of interest.

## Publisher’s note

All claims expressed in this article are solely those of the authors and do not necessarily represent those of their affiliated organizations, or those of the publisher, the editors and the reviewers. Any product that may be evaluated in this article, or claim that may be made by its manufacturer, is not guaranteed or endorsed by the publisher.

## Supplementary material

The Supplementary material for this article can be found online at: https://www.frontiersin.org/articles/10.3389/fmicb.2022.1074513/full#supplementary-material
