# Supplementary material for: Development of a colloidal gold immunochromatographic assay strip using monoclonal antibody for rapid detection of porcine deltacoronavirus
Source: Front Microbiol. 2023 Jan 5;13:1074513. doi: 10.3389/fmicb.2022.1074513 (PMC9849564; doi:10.3389/fmicb.2022.1074513)
Supplement: Supplementary file 1 [file Data_Sheet_1.PDF]

Supplementary material

Supplementary Figure 1 PDCoV antigen was positive for samples NO.36-50, but negative for No.51 sample using the GIGA strip. Also, these samples were confirmed by RT-qPCR.

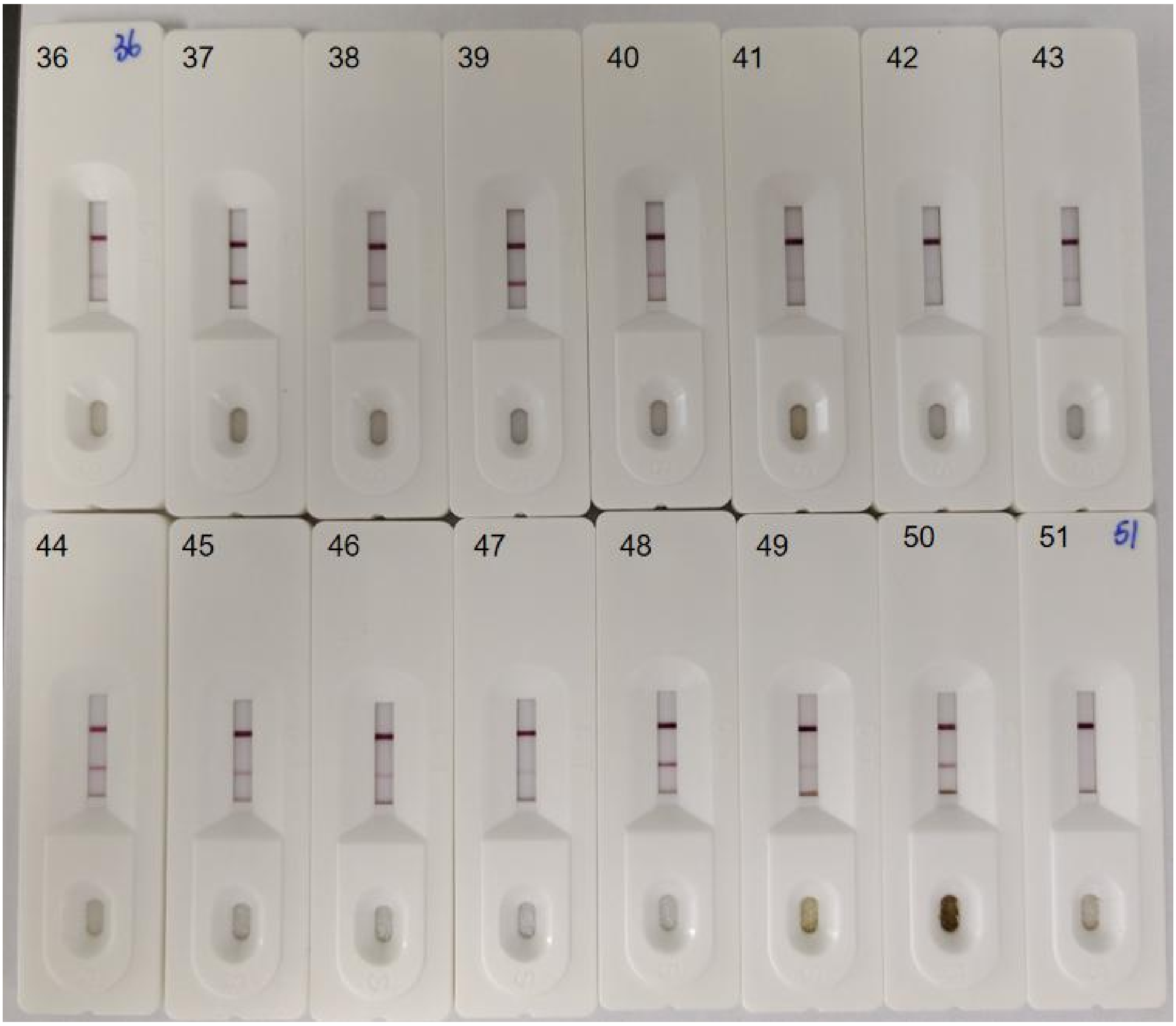

Supplementary Figure 1 The results of some fecal samples detected by using the GIGA strip
